# Factor Structure and Construct Validity of the Youth Psychopathic Traits Inventory and Its Shorten Version in Chinese Detained Boys

**DOI:** 10.3389/fpsyg.2019.01831

**Published:** 2019-08-07

**Authors:** Wendeng Yang, Xintong Zhang, Meng-Cheng Wang, Chuxian Zhong, Jie Luo, Yu Gao

**Affiliations:** ^1^Department of Psychology, Guangzhou University, Guangzhou, China; ^2^The Key Laboratory for Juveniles Mental Health and Educational Neuroscience in Guangdong Province, Guangzhou University, Guangzhou, China; ^3^The Center for Psychometrics and Latent Variable Modeling, Guangzhou University, Guangzhou, China; ^4^School of Psychology, Guizhou Normal University, Guiyang, China; ^5^Brooklyn College, The City University of New York, New York, NY, United States

**Keywords:** psychopathy, youth psychopathic traits inventory, factor structure, Chinese juveniles, incarcerated

## Abstract

The Youth Psychopathic Traits Inventory (YPI) was designed to assess psychopathic traits in adolescents. However, there exists limited evidence for the factor structure and psychometric properties of the YPI when used with Chinese detained juveniles. The present study aimed to examine the factor structure and construct validity of the YPI and its shortened version (YPI-S) in a sample of 607 Chinese 14- to 22-year-old detained boys (*M* = 17.15, *SD* = 1.09). Confirmatory factor analyses revealed a bifactor model which best fit the data at the subscale level for the YPI, and at the item level for the YPI-S. The internal consistency of the YPI and YPI-S scores ranged from marginal to good. Both the YPI and YPI-S total and factor scores correlated positively with the APSD, an alternative psychopathic measure, as well as with proactive and reactive aggression, and correlated negatively with affective and cognitive empathy. Overall, the YPI and YPI-S are shown to be practical and valid assessment tools to measure psychopathic traits in Chinese detained youths.

## Introduction

Psychopathy or psychopathic personality is a multifaceted personality disorder which has, to date, obtained considerable attention in both clinical and forensic settings. Generally, the psychopathic construct encompasses a constellation of interpersonal (e.g., manipulativeness, superficial charm, narcissism), affective (e.g., callousness, guiltlessness, lack of remorse) and behavioral traits (e.g., impulsivity, high sensation-seeking, dishonesty) (Cooke and Michie, 2001; Hare and Neumann, 2005). It has been noted that juveniles with a high measurement of psychopathic traits tend to display severe conduct problems and antisocial behavior, indulging in substance use and engaging in earlier criminal careers (e.g., Christian et al., 1997; Hare and Neumann, 2005). As psychopathic offenders, these youth exhibit associations with institutional violence (Brandt et al., 1997) and a resistance to rehabilitation efforts, resulting in a higher prevalence of recidivism (Hare, 2003; Rice and Harris, 2013).

The primary difficulty highlighted in current research is how to measure these psychopathic traits as precisely as possible. Youth incarceration facilities often lack the financial resources necessary for expert practitioners to carry out comprehensive evaluations on all potentially psychopathic adolescents (Colins et al., 2012), nor do they generally have access to reliable diagnostic information from parents and teachers (Kroll et al., 2002; Colins et al., 2008). Self-report assessments which measure the psychopathic construct, most notably the self-report version of the Antisocial Process Screening Device (APSD-SR; Frick and Hare, 2001) and the YPI (Andershed et al., 2002b), address these concerns, and have been useful tools for researchers and clinicians working with detained youths. Self-report instruments use less time and cost for the assessment especially in a large sample, making it easier to find individuals with particular subjective dispositions (e.g., guiltlessness) or intentions in their actions (e.g., manipulativeness) (Andershed et al., 2002a; Loney et al., 2003; Colins et al., 2014). However, the one issue with using psychopathic self-reports is that individuals with high psychopathic traits may intentionally distort their assessment responses, to make their traits seem either more or less socially desirable (Colins et al., 2017; Pechorro et al., 2017).

The YPI was a promising self-report tool that was first developed to assess psychopathic personality traits in community samples of adolescents. Soon, however, the YPI was also validated across various samples including juvenile offenders. The items of the YPI have the unique advantage of being designed to be neutral or attractive characteristics rather than deficits, which encourage psychopathic individuals admit to having such traits, even if these traits are generally regarded as being socially undesirable or malignant (Andershed et al., 2002a). The YPI questionnaire covers 10 subscales (e.g., lying, callousness, irresponsibility) which combine into three core psychopathic personality components: interpersonal (or grandiose-manipulative), affective (or callous-unemotional), and behavior/lifestyle (or impulsive-irresponsible) (Andershed et al., 2002b). These three features align with Cooke and Michie’s three-factor model of psychopathy (Cooke and Michie, 2001).

Emerging research on the YPI has shown promising psychometric properties among non-referred (Andershed et al., 2002a,b, 2007), delinquent (Skeem and Cauffman, 2003) and forensic adolescents (Poythress et al., 2006). Exploratory and confirmatory factor analyses have demonstrated that a three-factor model conducted on the 10 subscales best fit most samples (e.g., Andershed et al., 2002a; Poythress et al., 2006; Declercq et al., 2009). Still, a bifactor model with a general factor is also viewed as an acceptable alternative framework for psychopathy, and has been recently proposed as effective in a sample of 596 French-speaking community and institutionalized adolescents (Pihet et al., 2014), as well as a sample of 2,874 Dutch adolescent students (Zwaanswijk et al., 2016). Likewise, in Chinese community youth, Wang et al. (2017) compared the various factor structures tested in previous literature and found the best fitting bifactor model for the Chinese-language versions of the YPI at the subscale level. According to existing research (Pihet et al., 2014; Pechorro et al., 2015; Wang et al., 2017), the internal consistency using Cronbach’s α of the YPI total and three-factor scores was consistently high to acceptable (between 0.70 and 0.93 in the community sample, and above 0.90 in incarcerated youth sample). However, some of the 10 subscales showed extremely low internal consistency; for example, the Cronbach’s αs of callousness and unemotionality were only 0.40 and 0.47, respectively, in Chinese adolescents (Wang et al., 2017). As to convergent validity, both the YPI total score and the three-factor scores showed the expected associations with alternative psychopathic measures, such as the APSD (Skeem and Cauffman, 2003; Andershed et al., 2007; Wang et al., 2017) and Levenson’s Self-Report Psychopathy Scale (LSRP; Wang et al., 2018). The criterion validity of the YPI was also supported by significantly positive associations with aggression, conduct problems, and early onset of delinquency (e.g., Poythress et al., 2006; Veen et al., 2011; Pechorro et al., 2015; Wang et al., 2017), and negative associations with anxiety and depression (e.g., Andershed et al., 2002a; Skeem and Cauffman, 2003; Poythress et al., 2006).

The Youth Psychopathic Traits Inventory – Short Version (YPI-S; van Baardewijk et al., 2010) is a time-saving version of the full YPI for use in multidisciplinary studies (Colins et al., 2012). A total of 18 items were selected through a stepwise selection process using a series of exploratory and confirmatory factor analyses and content-related arguments. The YPI-S examines the three main factors instead the 10 subscales measured in the YPI, but measures the same interpersonal, affective, and behavioral traits of the psychopathy construct (Colins et al., 2012). Studies regarding the three-factor model of the YPI-S (e.g., van Baardewijk et al., 2010; Colins et al., 2012; Pechorro et al., 2015) have supported this structure, with most studies finding the YPI-S identical to the factor structure of the full-length YPI, except for one: Wang et al. (2017) found that a bifactor model achieved an optimal model fit at the item level in a Chinese community sample, but that this finding still warranted a larger body of evidence. Overall, the internal consistency of the YPI-S total and its factors were generally modest to good, showing a high convergence with the original YPI as well as with other psychopathic measures (e.g., LSRP; Wang et al., 2018), conduct problems, and criminal variables (e.g., self-reported offending, aggression; Pechorro et al., 2015, 2017). There is evidence that, in spite of both versions of the YPI having similar psychometric measurements, the original YPI shows a better performance record than the YPI-S, particularly in consideration of the assessment time (Wang et al., 2017).

In conclusion, both the YPI and YPI-S are the current most promising instruments used to assess a psychopathic personality. To our knowledge, only one study (Wang et al., 2017) has systematically examined the factor structure and psychometric properties of the Chinese version of the YPI and YPI-S, but this study used a sample of Chinese community adolescents. Assessment using Chinese detained youth has been an unknown before now. Moreover, both the number of juvenile crimes or criminal nature of the case has presented a more serious and younger age trend from relevant statistical data show in recent years. Investigation of the psychopathic traits of incarcerated adolescents may benefit the improvement in the system of current criminal penalty.

### The Current Study

The main aim of the current study is to examine the psychometric properties of the Chinese version of both the YPI and its abbreviated form, the YPI-S, among a forensic sample of incarcerated male youths. This investigation had three specific purposes: the first was to conduct a confirmatory factor analysis (CFA) to compare various factor structures proposed in previous studies (e.g., Andershed et al., 2002a; van Baardewijk et al., 2010; Wang et al., 2017). In light of prior findings (e.g., Pechorro et al., 2017; Wang et al., 2017), it is predicted that the bifactor structure of both the YPI and YPI-S would be replicated both at the item and subscale levels. The second purpose was to test the internal consistency of the YPI and YPI-S total and factor scores. The expectation was that the YPI-S would maintain satisfactory internal consistency in comparison with the original version, despite the removal of nearly two thirds of the items from the YPI. The third purpose was to examine the convergent and criterion-related validity of the YPI and YPI-S scores. Zero-order and partial correlations were computed to scrutinize the associations of the YPI and YPI-S total and factor scores with relevant external variables. Specifically, it was hypothesized that the YPI and YPI-S would associate positively with the psychopathy construct (e.g., callous-unemotional traits, narcissism), and with reactive and proactive aggression, yet they would largely show moderate or non-significant correlations with affective and cognitive empathy. Finally, the YPI-S was expected to be found to be an informative alternative to the original YPI format, particularly when being administered to detained Chinese male youths.

## Materials and Methods

### Participants

Male inmates recruited from the Guangdong Juvenile Detention Center voluntarily agreed to participate in this study (*N* = 614). Data of seven participants was excluded due to the fact that they replied to less than 50% of the questionnaire items. A total of 607 participants (*M* = 17.51 years, *SD* = 1.09 years, age range = 14–22 years) were Han (86.0%), and 13.3% were members of a mix of other ethnic minorities. More than half of the juvenile subjects had been first detained before they were 16 years old (*M* = 15.49 years, *SD* = 0.87 years), and most of them (66.9%) had been convicted of robbery (*N* = 406), followed by physical assault (*N* = 70, 11.5%), and sexual assault (*N* = 50, 8.2%). Approximately 76.0% of the subjects were from nuclear families (*N* = 461), but a few came from single-child families (*N* = 110, 18.1%).

### Procedure

Prior to completing the assessments, a formal agreement was made with the relevant staff of the juvenile detention center, and written informed consent was obtained from the adolescents as well as from their parents or legal guardian. The self-reported questionnaires were administered digitally in a classroom setting, in a session that lasted approximately 40 min under the supervision of specially trained research assistants. Participants were informed that completing the questionnaires was voluntary, and that they were allowed to ask for clarification if they did not understand any part of the questionnaires.

### Measures

#### The Youth Psychopathic Traits Inventory (YPI)

The YPI is a 50-item self-report questionnaire designed to evaluate the core personality traits of the psychopathic personality constellation. The YPI assesses each psychopathic trait using five items to form 10 different subscales (Andershed et al., 2002b). These subscales manifest in a three-factor model comprising the Grandiose-Manipulative factor (GM; 20 items), the callous-unemotional factor (CU; 15 items), and the impulsive-irresponsible factor (II; 15 items). The GM factor consists of the dishonest charm, grandiosity, lying, and manipulation subscales; the CU factor consists of the callousness, unemotional, and remorselessness subscales; the II factor consists of the impulsivity, thrill-seeking, and irresponsibility subscales. Participants rated statements on a four-point scale ranging from 1 (“Does not apply at all”) to 4 (“Applies very well”).

The YPI-S is an 18-item, shortened version of the original YPI, but aims to assess higher-order factors using six items, which consists of the GM, CU, and II factors. All items of the YPI-S were selected from the original YPI. Both the YPI and its abbreviated version are scored by simply adding together all answers specific to the relevant factor, with higher scores showing a serious presence of psychopathic characteristics. In the current study, participants completed the Chinese validation of the YPI which had been translated and validated by Wang and colleagues (Wang et al., 2017).

#### The Antisocial Process Screening Device – Self-Report Version (APSD-SR)

The APSD-SR is a multidimensional 20-item assessment that measures antisocial behaviors and psychopathic traits in youth. Each item is rated on a three-point Likert scale from 0 (“Not at all true”) to 2 (“Definitely true”). The total score, as well as the score for each factor, is obtained by summing up the items relevant to each factor. A preferred three-factor structure constitutes a seven-item narcissistic (Nar) factor, a five-item impulsivity (Imp) factor, and a six-item CU factor. The Chinese validation of the APSD-SR (Wang et al., 2015) was used. In line with previous studies with justice-involved youths (e.g., Murrie and Cornell, 2002; Pardini et al., 2003), internal consistency for the total score was satisfactory (α = 0.715), but were modest to weak for the Nar, Imp and CU: 0.557, 0.613, and 0.446, respectively.

#### The Basic Empathy Scale (BES)

The BES (Jolliffe and Farrington, 2006; Geng et al., 2012) is a concise, coherent self-report measure made up of 20 items, designed to evaluate empathy in adolescents. Two distinct subscales have been identified: affective empathy is assessed by 11 items, and cognitive empathy is assessed by nine items. Each item is scored on a five-point Likert scale ranging from 1 (“Strongly disagree”) to 5 (“Strongly agree”). Scores of relevant items are added together to form measures of affective or cognitive empathy, while also producing an overall total empathy score. In the present study, the Cronbach’s αs for BES total, the affective, and the cognitive empathy scales were 0.737, 0.679, and 0.755, respectively.

#### The Reactive-Proactive Aggression Questionnaire (RPQ)

The RPQ (Raine et al., 2006; Fung et al., 2009) is a self-report questionnaire comprising 23 items, which yields both reactive (11 items) and proactive (12 items) aggression scores for youth and young adults. Each item is scored on a three-point scale from 0 (“Never”) to 2 (“Often”). Higher scores demonstrate higher levels of aggression. The Cronbach’s αs were: RPQ total = 0.936, proactive dimension = 0.900, and reactive dimension = 0.874.

#### Data Analysis Strategy

All analyses were carried out with SPSS (IBM, SPSS version 19, 2010) and Mplus 7.4 (Muthén and Muthén, 1998–2015). The factor structures of the Chinese language version of the YPI at both the item and subscale levels, as well as the YPI-S at the item level, were assessed using CFA performed with Mplus 7.4, in line with findings from Wang et al. (2017). The robust weighted least-squares with a mean and variance adjustment (WLSMV) estimator was adopted to account for the categorical nature of the responses (Flora and Curran, 2004), and the robust maximum likelihood estimator (MLR) was also used to address the possibility of non-normal distributions of YPI subscales. Model fit indices consisted of chi-square (χ^2^), root mean square error of approximation (RMSEA), the Tucker-Lewis index (TLI), and the comparative fit index (CFI). Conventional guidelines suggest that a value of 0.90 or higher for both the TLI and CFI, and an RMSEA value of 0.05 or smaller manifests a satisfactory model fit, and that ≤0.80 indicates an acceptable fit (Kline, 2010).

As a follow-up to CFA, the explained common variance (ECV) was calculated to assess the degree of unidimensionality of the YPI and YPI-S bifactor models, comparing the common variance with the variance as explained by specific overall factors. As a general index of unidimensionality in a bifactor model (Revelle and Wilt, 2013), an ECV value that approaches 1 (e.g., >0.70) implies that the general factor loadings are partly equal to those obtained by the estimate of a one-dimensional model (Rodriguez et al., 2015). Coefficient omega (ω) is a reliability estimate based on the factor loadings and was computed to estimate the proportion of variance in the observed YPI and YPI-S total scores, which “modeled” sources of common variance results (Reise et al., 2013). Coefficient omega hierarchical (ω_*H*_) and the omega hierarchical subscale (ω_*HS*_) are the alternative indices of ω used to clarify what role the various sources play in determining composite score variance (Reise, 2012). The ω_*H*_ is used for the general factor, without variability of group factors, while the ω_*HS*_ is used for a group factor without the influence of all other group and general factors (Brunner et al., 2012; Reise, 2012). A high ω_*H*_ reflects a large proportion of reliable variance attributable to a single common source, thus validating a unidimensional model. Meanwhile, a multifaceted psychopathy construct is reflected by higher ω_*HS*_ values (Reise et al., 2013).

Cronbach’s αs were computed to assess the internal consistency of the models. Ranges of measures are as follows: <0.60 = insufficient; 0.60–0.69 = marginal; 0.70–0.79 = acceptable; 0.80–0.89 = good; and 0.90 or higher = excellent (Barker et al., 1994). To account for the short length of some of the models, mean inter-item correlations (MIC) were computed as an alternative measure of internal consistency. A MIC value between 0.15 and 0.50 indicates a satisfactory internal consistency (Clark and Watson, 1995).

In terms of convergent and criterion-related validity, zero-order and partial correlations with the shared variance of the other subscales controlled, were performed on the YPI and YPI-S scores, and compared against the APSD-SR scores, the BES scores, and the RPQ scores. A correlation (*r*) of ≤0.29 is considered low; an *r* from 0.30 to 0.49 is considered medium; and an *r* ≥ 0.50 is considered large (Cohen, 1988). Consistent with Wang et al. (2017), the method proposed by Dunn and Clark (1969) was conducted to compare the strength of the correlations link between the original YPI to criterion measures with that of the YPI-S (see Steiger, 1980 for more details).^1^ Descriptive information for all scales measured in the current study is found in Table 1.

**TABLE 1:** Descriptive statistics and internal consistency for all scales included.

	***M*(*SD*)**	**Range**	**α**	**MIC**	**N of Items**
**YPI (*N* = 607)**					
YPI total score	87.33(18.92)/80.18(18.75)	51–191/48–188	0.914/0.921	0.184/0.207	50/47
YPI GM	30.42(8.40)	20–80	0.877	0.273	20
Dishonest charm	7.44(2.60)	5–20	0.721	0.360	5
Grandiosity	8.29(2.89)	5–20	0.732	0.358	5
Lying	7.50(2.68)	5–20	0.761	0.389	5
Manipulation	7.28(2.46)	5–20	0.689	0.323	5
YPI CU	28.80(5.75)/21.67(5.38)	16–57/12–48	0.664/0.723	0.124/0.185	15/12
Remorselessness	8.18(2.77)	5–20	0.619	0.248	5
Unemotional	9.99(2.68)	5–20	0.512	0.176	5
Callousness	10.63(2.46)/3.50(1.30)	5–20/2–8	0.343/0.109	0.105/0.070	5/2
YPI II	28.67(8.41)	15–60	0.870	0.310	15
Thrill-seeking	10.07(3.49)	5–20	0.786	0.420	5
Impulsivity	9.99(3.33)	5–20	0.721	0.351	5
Irresponsibility	8.59(2.89)	5–20	0.634	0.263	5
**YPI-S (*N* = 607)**					
YPI-S total score	30.71(7.14)	18–72	0.786	0.179	18
YPI-S GM	8.80(2.75)	6–24	0.695	0.294	6
YPI-S CU	10.71(3.04)	6–24	0.545	0.174	6
YPI-S II	11.30(3.68)	6–24	0.758	0.340	6
**APSD (*N* = 607)**					
APSD total score	10.55(4.83)	1–30	0.715	0.114	20
Narcissism	3.48(2.29)	0–14	0.557	0.157	7
Impulsivity	3.36(2.42)	0–10	0.613	0.240	5
Callous-Unemotional	3.68(2.03)	0–11	0.446	0.129	6
**BES (*N* = 607)**					
BES total score	68.25(8.79)	42–99	0.737	0.124	20
Affective empathy	34.68(6.05)	15–54	0.679	0.159	11
Cognitive empathy	33.49(5.32)	19–45	0.755	0.260	9
**RPQ (*N* = 607)**					
RPQ total score	11.82(9.14)	0–46	0.936	0.391	23
Proactive aggression	5.02(5.00)	0–24	0.900	0.434	12
Reactive aggression	6.83(4.63)	0–22	0.874	0.388	11

*YPI, the Youth Psychopathic Traits Inventory; YPI-S, the Youth Psychopathic Traits Inventory – Short Version; GM, grandiose-manipulative; CU, callous-unemotional; II, impulsive-irresponsible; APSD, the self-report Antisocial Process Screening Device; BES, the Basic Empathy Scale; RPQ, the Reactive-Proactive Aggression Questionnaire; MIC, mean inter-item correlation. Results of the YPI total, CU factor and callousness subscale without the reversed items are presented after the tab.*

## Results

### Confirmatory Factor Analysis

Goodness-of-fit indices for the competitive models are illustrated in Tables 2, 3. At the YPI item level, the model showing the best fit was the bifactor structure (χ^2^ = 2855.949, *df* = 1125; CFI = 0.854, TLI = 0.841, RMSEA = 0.050). Note that the three reversed scored CU items (i.e., “It’s important to me not to hurt others’ emotions or feelings,” “I often become sad or moved by watching sad things on TV or film,” and “I usually become sad when I see others sad or crying”) had poor factor loadings in all models, as shown in Table 4. Moreover, Pearson correlations of the composite scores of the above-mentioned items with the other nine factors ranged from −0.003 to −0.053, so these three items were dropped in further examinations. However, the fit of the modified correlated three-factor model (χ^2^ = 3250.164, *df* = 1031; CFI = 0.806, TLI = 0.797, RMSEA = 0.060), as well as the modified bifactor model (χ^2^ = 2600.084, *df* = 987; CFI = 0.859, TLI = 0.846, RMSEA = 0.052), remained poor, which was in line with Wang et al. (2017).

**TABLE 2:** Model fits indices for the YPI without the three reversed items at the item and subscale levels, and for the YPI-S at the item level.

	**WLSMVχ^2^/MLRχ^2^**	***df***	**CFI**	**TLI**	**RMSEA(90% CI)**
YPI item level-1F	4324.130^∗∗^	1034	0.713	0.700	0.072(0.070, 0.075)
YPI item level-correlated 3F	3250.164^∗∗^	1031	0.806	0.797	0.060(0.057, 0.062)
YPI item level-uncorrelated 3F	7522.651^∗∗^	1034	0.433	0.408	0.102(0.100, 0.104)
YPI item level-3FBF	2600.084^∗∗^	987	0.859	0.846	0.052(0.049, 0.054)
YPI item level-10F	2497.419^∗∗^	989	0.868	0.856	0.050(0.048, 0.053)
YPI scale level-1F	407.019^∗∗^	35	0.816	0.763	0.132(0.121, 0.144)
YPI scale level-correlated 3F	109.083^∗∗^	32	0.962	0.946	0.063(0.050, 0.076)
YPI scale level-uncorrelated 3F	583.016^*^	35	0.729	0.651	0.161(0.149, 0.172)
YPI scale level-3FBF	91.449^∗∗^	25	0.967	0.941	0.066(0.052, 0.081)
YPI-S item level-1F	1048.767^∗∗^	135	0.717	0.679	0.106(0.100, 0.112)
YPI-S item level-correlated 3F	418.517^∗∗^	132	0.911	0.897	0.060(0.053, 0.066)
YPI-S item level-uncorrelated 3F	1169.394^∗∗^	135	0.680	0.637	0.112(0.106, 0.118)
YPI-S-item level-3FBF	291.912^∗∗^	117	0.946	0.929	0.050(0.043, 0.057)

*YPI, the Youth Psychopathic Traits Inventory; YPI-S, the Youth Psychopathic Traits Inventory – Short Version; WLSMV, the weighted least squares estimator for model on item level; MLR, Robust Maximum Likelihood Estimator for model on scale level; χ^2^, chi-square test; df, degrees of freedom; CFI, comparative fit index; TLI, Tucker-Lewis index; RMSEA, the root-mean-square error of approximation; CI, confidence interval; 1F, one-factor model; 3F, three-factor model; 10F, 10-factor model; 3FBF, three-factor bifactor model. ^∗∗^*p* < 0.001.*

**TABLE 3:** Model fits indices for the YPI at the item and subscale levels and for the YPI-S at the item level.

	**WLSMVχ^2^/MLRχ^2^**	***df***	**CFI**	**TLI**	**RMSEA(90% CI)**
YPI item level-1F	4713.276^∗∗^	1175	0.701	0.688	0.070(0.068, 0.073)
YPI item level-correlated 3F	3695.888^∗∗^	1172	0.786	0.777	0.060(0.057, 0.062)
YPI item level-correlated 3F–dropped 3 items	3250.164^∗∗^	1031	0.806	0.797	0.060(0.057, 0.062)
YPI item level-uncorrelated 3F	7904.385^∗∗^	1175	0.431	0.406	0.097(0.095, 0.099)
YPI item level-3FBF	2855.949^∗∗^	1125	0.854	0.841	0.050(0.048, 0.053)
YPI item level-10F	2984.799^∗∗^	1130	0.843	0.830	0.052(0.050, 0.054)
YPI scale level-1F	471.979^∗∗^	35	0.817	0.765	0.143(0.132, 0.155)
YPI scale level-correlated 3F	115.971^∗∗^	32	0.957	0.940	0.066(0.053, 0.079)
YPI scale level-uncorrelated 3F	589.047^∗∗^	35	0.718	0.637	0.161(0.150, 0.173)
YPI scale level-3FBF	96.595^∗∗^	25	0.964	0.934	0.069(0.055, 0.083)
YPI-S item level-1F	1048.767^∗∗^	135	0.717	0.679	0.106(0.100, 0.112)
YPI-S item level-correlated 3F	418.517^∗∗^	132	0.911	0.897	0.060(0.053, 0.066)
YPI-S item level-uncorrelated 3F	1169.394^∗∗^	135	0.680	0.637	0.112(0.106, 0.118)
YPI-S-item level-3FBF	291.912^∗∗^	117	0.946	0.929	0.050(0.043, 0.057)

*YPI, the Youth Psychopathic Traits Inventory; YPI-S, the Youth Psychopathic Traits Inventory – Short Version; WLSMV, the weighted least squares estimator for model on item level; MLR, Robust Maximum Likelihood Estimator for model on scale level; χ^2^, chi-square test; df, degrees of freedom; CFI, comparative fit index; TLI, Tucker-Lewis index; RMSEA, the root-mean-square error of approximation; CI, confidence interval; 1F, one-factor model; 10F, ten-factor model; 3FBF, three-factor bifactor model. ^∗∗^*p* < 0.001.*

**TABLE 4:** Standardized factor loadings of items for three models of YPI (1F/correlated 3F/uncorrelated 3F/10F).

	**1F**	**Correlated 3F**	**Uncorrelated 3F**	**10F**
**Grandiose-Manipulative factor**				
**Dishonest charm**				
YPI6-It’s easy for me to charm …	0.575^∗∗^	0.629^∗∗^	0.623^∗∗^	0.661^∗∗^
YPI14-I have the ability to con …	0.724^∗∗^	0.778^∗∗^	0.784^∗∗^	0.818^∗∗^
YPI27-When someone asks me …	0.638^∗∗^	0.690^∗∗^	0.667^∗∗^	0.726^∗∗^
YPI33-Pretty often I act charming …	0.552^∗∗^	0.600^∗∗^	0.581^∗∗^	0.631^∗∗^
YPI38-When I need to, I use my …	0.611^∗∗^	0.666^∗∗^	0.690^∗∗^	0.699^∗∗^
**Grandiosity**				
YPI10-I’m better than everyone …	0.283^∗∗^	0.349^∗∗^	0.460^∗∗^	0.590^∗∗^
YPI19-I have talents that go far …	0.409^∗∗^	0.478^∗∗^	0.562^∗∗^	0.748^∗∗^
YPI30-The world would be a …	0.486^∗∗^	0.537^∗∗^	0.524^∗∗^	0.772^∗∗^
YPI37-I’m more important and …	0.368^∗∗^	0.431^∗∗^	0.508^∗∗^	0.684^∗∗^
YPI41-I am destined to become …	0.367^∗∗^	0.420^∗∗^	0.468^∗∗^	0.640^∗∗^
**Lying**				
YPI7-It’s fun to make up stories …	0.638^∗∗^	0.691^∗∗^	0.704^∗∗^	0.750^∗∗^
YPI24-Sometimes I lie for no …	0.607^∗∗^	0.654^∗∗^	0.629^∗∗^	0.716^∗∗^
YPI43-Sometimes I find myself …	0.667^∗∗^	0.717^∗∗^	0.660^∗∗^	0.784^∗∗^
YPI47-I like to spice up and …	0.617^∗∗^	0.663^∗∗^	0.622^∗∗^	0.726^∗∗^
YPI50-I’ve often gotten into …	0.531^∗∗^	0.578^∗∗^	0.567^∗∗^	0.627^∗∗^
**Manipulation**				
YPI11-I can make people believe …	0.434^∗∗^	0.500^∗∗^	0.553^∗∗^	0.498^∗∗^
YPI15-I am good at getting people …	0.695^∗∗^	0.760^∗∗^	0.810^∗∗^	0.751^∗∗^
YPI20-It’s easy for me to …	0.596^∗∗^	0.652^∗∗^	0.603^∗∗^	0.644^∗∗^
YPI31-To get people to do what …	0.765^∗∗^	0.820^∗∗^	0.767^∗∗^	0.807^∗∗^
YPI46-It has happened that I’ve …	0.618^∗∗^	0.666^∗∗^	0.648^∗∗^	0.656^∗∗^
**Callous-Unemotional factor**				
**Remorselessness**				
YPI8-I have the ability not to …	0.461^∗∗^	0.533^∗∗^	0.553^∗∗^	0.526^∗∗^
YPI21-I seldom regret things I …	0.470^∗∗^	0.539^∗∗^	0.500^∗∗^	0.531^∗∗^
YPI28-When someone finds out …	0.625^∗∗^	0.717^∗∗^	0.523^∗∗^	0.707^∗∗^
YPI44-To feel guilty and remorseful …	0.466^∗∗^	0.538^∗∗^	0.554^∗∗^	0.531^∗∗^
YPI48-To feel guilt and regret …	0.485^∗∗^	0.565^∗∗^	0.673^∗∗^	0.556^∗∗^
**Unemotionality**				
YPI2- I usually feel calm when …	0.350^∗∗^	0.402^∗∗^	0.406^∗∗^	0.459^∗∗^
YPI25-To be nervous and worried …	0.446^∗∗^	0.518^∗∗^	0.586^∗∗^	0.576^∗∗^
YPI36-What scares others usually …	0.379^∗∗^	0.436^∗∗^	0.428^∗∗^	0.494^∗∗^
YPI39-I don’t understand how …	0.394^∗∗^	0.453^∗∗^	0.458^∗∗^	0.497^∗∗^
YPI45-I don’t let my feelings affect …	0.118^*^	0.149^∗∗^	0.281^∗∗^	0.179^∗∗^
**Callousness**				
YPI12-I think that crying is a sign …	0.306^∗∗^	0.355^∗∗^	0.399^∗∗^	0.233^*^
YPI17-When other people have …	0.415^∗∗^	0.477^∗∗^	0.480^∗∗^	0.313^*^
YPI23-It’s important to me not … (reversed)	0.048	0.049	−0.024	0.032
YPI35-I often become sad or … (reversed)	−0.007	−0.006	0.047	−0.003
YPI49-I usually become sad … (reversed)	−0.040	−0.040	0.032	−0.026
**Impulsive-Irresponsible factor**				
**Thrill-seeking**				
YPI1- I like to be where exciting …	0.632^∗∗^	0.694^∗∗^	0.702^∗∗^	0.742^∗∗^
YPI4-I get bored quickly when …	0.566^∗∗^	0.631^∗∗^	0.615^∗∗^	0.678^∗∗^
YPI22-I like to do things just for …	0.692^∗∗^	0.761^∗∗^	0.788^∗∗^	0.809^∗∗^
YPI29-I get bored quickly by …	0.581^∗∗^	0.647^∗∗^	0.630^∗∗^	0.696^∗∗^
YPI42-I like to do exciting and …	0.762^∗∗^	0.844^∗∗^	0.791^∗∗^	0.905^∗∗^
**Impulsivity**				
YPI3-I prefer to spend my money …	0.571^∗∗^	0.638^∗∗^	0.643^∗∗^	0.702^∗∗^
YPI9-If I get the chance to do …	0.573^∗∗^	0.643^∗∗^	0.656^∗∗^	0.709^∗∗^
YPI18-It often happens that I …	0.620^∗∗^	0.698^∗∗^	0.740^∗∗^	0.768^∗∗^
YPI26-If I get the chance to do …	0.460^∗∗^	0.502^∗∗^	0.427^∗∗^	0.540^∗∗^
YPI32-It often happens that I do …	0.637^∗∗^	0.714^∗∗^	0.756^∗∗^	0.787^∗∗^
**Irresponsibility**				
YPI5-I have probably skipped …	0.534^∗∗^	0.602^∗∗^	0.619^∗∗^	0.651^∗∗^
YPI13-If I won a lot of money in …	0.487^∗∗^	0.540^∗∗^	0.505^∗∗^	0.581^∗∗^
YPI16-I have often been late to …	0.555^∗∗^	0.624^∗∗^	0.640^∗∗^	0.678^∗∗^
YPI34-It has happened several …	0.380^∗∗^	0.417^∗∗^	0.364^∗∗^	0.453^∗∗^
YPI40-I often don’t/didn’t have …	0.505^∗∗^	0.565^∗∗^	0.541^∗∗^	0.611^∗∗^

*YPI, the Youth Psychopathic Traits Inventory; 1F, one-factor model; 3F, three-factor model; 10F, ten-factor model. ^∗∗^*p* < 0.001.*

At the subscale level, the bifactor model provided adequate fit indices (χ^2^ = 91.449, *df* = 25; CFI = 0.967, TLI = 0.941, RMSEA = 0.066).^2^ All items loaded on the general factor were in the range of 0.083 to 0.793 (see Table 5). The ω_*HS*_ for the general factor, GM, CU, and II were 0.785, 0.245, 0.163, and 0.293 with ECV being 69.1%.

**TABLE 5:** Standardized factor loadings for the YPI bifactor model at the subscale level.

**Factor**	**GM**	**CU**	**II**	**General factor**
**Grandiose-Manipulative factor (GM)**				
Dishonest charm	0.407^∗∗^/0.406^∗∗^			0.668^∗∗^/0.670^∗∗^
Grandiosity	0.320^∗∗^/0.329^∗∗^			0.389^∗∗^/0.384^∗∗^
Lying	0.264^*^/0.255			0.664^∗∗^/0.669^∗∗^
Manipulation	0.588^∗∗^/0.575^∗∗^			0.708^∗∗^/0.716^∗∗^
**Callous-Unemotional factor (CU)**				
Remorselessness		0.196/2.367		0.729^∗∗^/0.728^∗∗^
Unemotional		0.415^*^/0.037		0.548^∗∗^/0.542^∗∗^
Callousness		0.327^*^/0.037		0.404^∗∗^/0.209^∗∗^
**Impulsive-Irresponsible factor (II)**				
Thrill-seeking			0.364^∗∗^/0.368^∗∗^	0.678^∗∗^/0.676^∗∗^
Impulsivity			0.550^∗∗^/0.553^∗∗^	0.638^∗∗^/0.635^∗∗^
Irresponsibility			0.492^∗∗^/0.495^∗∗^	0.598^∗∗^/0.595^∗∗^
ω	0.825	0.687	0.837	0.901
ω_*H*_/ω_*HS*_	0.245	0.163	0.293	0.785

YPI, the Youth Psychopathic Traits Inventory; GM, grandiose-manipulative; CU, callous-unemotional; II, Impulsive-Irresponsible. Results of the YPI without the reversed items are presented before the tab. ^*^p < 0.01, ^∗∗^*p* < 0.001.

At the YPI-S item level, none of the fit indices were acceptable for the one-factor model or the uncorrelated three-factor model. For the correlated three-factor model, the fit indices were marginally acceptable (TLI = 0.897) to acceptable (CFI = 0.911, RMSEA = 0.060), whereas all indices for the bifactor model in the present sample met the criteria of good fit (χ^2^ = 291.912, *df* = 117; CFI = 0.946, TLI = 0.929, RMSEA = 0.050). The factor loadings on the general factor for GM items, CU items, and II items varied from 0.193 to 0.635, from 0.129 to 0.557, and from 0.323 to 0.510, respectively. However, on the group factor (i.e., CU), the loadings of CU items ranged only from −0.052 to 0.388 (see Table 6). Furthermore, the ECV was dominated by the general factor at 48.7%. The ω_*HS*_ for the general factor, GM, CU, and II were 0.668, 0.489, 0.136, and 0.448.

**TABLE 6:** Standardized factor loadings for the YPI-S bifactor model at the item level.

**Item**	**GM**	**CU**	**II**	**General factor**
**GM**				
YPI14-I have the ability to con …	0.528^∗∗^			0.635^∗∗^
YPI15-I am good at getting people …	0.536^∗∗^			0.514^∗∗^
YPI19-I have talents that go far …	0.627^∗∗^			0.193^∗∗^
YPI20-It’s easy for me to …	0.420^∗∗^			0.499^∗∗^
YPI38-When I need to, I use my …	0.440^∗∗^			0.513^∗∗^
YPI41-I am destined to become …	0.503^∗∗^			0.211^∗∗^
**CU**				
YPI12-I think that crying is a sign …		0.388^∗∗^		0.338^∗∗^
YPI17-When other people have …		−0.068		0.519^∗∗^
YPI25-To be nervous and worried …		0.622^∗∗^		0.515^∗∗^
YPI39-I don’t understand how …		−0.052		0.511^∗∗^
YPI44-To feel guilty and remorseful …		0.200^*^		0.557^∗∗^
YPI45-I don’t let my feelings affect …		0.210^*^		0.129^*^
**II**				
YPI5-I have probably skipped …			0.341^∗∗^	0.481^∗∗^
YPI9-If I get the chance to do …			0.482^∗∗^	0.465^∗∗^
YPI18-It often happens that I …			0.760^∗∗^	0.413^∗∗^
YPI29-I get bored quickly by …			0.282^∗∗^	0.510^∗∗^
YPI32-It often happens that I do …			0.759^∗∗^	0.400^∗∗^
YPI34-It has happened several …			0.218^∗∗^	0.323^∗∗^
ω	0.834	0.667	0.821	0.882
ω_*H*_/ω_*HS*_	0.489	0.136	0.448	0.668

YPI-S, the Youth Psychopathic Traits Inventory – Short Version; GM, grandiose-manipulative; CU, callous-unemotional; II, impulsive-irresponsible. ^*^p < 0.01, ^∗∗^*p* < 0.001.

### Internal Consistency and Inter-Correlations

Table 1 displays the internal consistency of the YPI and YPI-S. Although the Cronbach’s αdemonstrated satisfactory internal consistency for the YPI total score (0.914), the α values for approximately half of the subscales, particularly callousness (α = 0.343; MIC = 0.105), were below the conventionally recommended criterion value of 0.70. In a three-factor model, the internal consistency of the YPI was good, except for the CU (α = 0.664; MIC = 0.124). Excluding the reversed items, the Cronbach’s α of the CU factor increased to 0.713 and MIC to 0.185, but this was only mirrored in two items included in the callousness subscale (i.e., “I think that crying is a sign of weakness, even if no one sees you” and “When other people have problems, it is often their own fault, therefore, one should not help them”). This resulted in a decrease in both α (0.109) and MIC (0.070). Table 7 shows that the inter-factor correlations of the YPI when three items were deleted were: *r*_*GM–CU*_ = 0.591, *r*_*GM–II*_ = 0.522, and *r*_*CU–II*_ = 0.571, and the correlations between the total score and factor scores were 0.814 (*r*_*Total–CU*_) to 0.855 (*r*_*Total–GM*_).

**TABLE 7:** Zero-order correlations between YPI without the reversed items and YPI-S.

	**YPI**				**YPI-S**			
**YPI**	**GM**	**CU**	**II**	**total**	**GM**	**CU**	**II**	**total**
GM	1							
CU	0.591^∗∗^	1						
II	0.522^∗∗^	0.571^∗∗^	1					
total	0.855^∗∗^	0.814^∗∗^	0.847^∗∗^	1				
**YPI-S**								
GM	**0.871^∗∗^**	0.514^∗∗^	0.382^∗∗^	0.712^∗∗^	1			
CU	0.463^∗∗^	**0.864^∗∗^**	0.420^∗∗^	0.649^∗∗^	0.401^∗∗^	1		
II	0.411^∗∗^	0.487^∗∗^	**0.887^∗∗^**	0.723^∗∗^	0.280^∗∗^	0.356^∗∗^	1	
total	0.746^∗∗^	0.821^∗∗^	0.785^∗∗^	**0.926^∗∗^**	0.700^∗∗^	0.768^∗∗^	0.777^∗∗^	1

*YPI, the youth psychopathic traits inventory; YPI-S, the youth psychopathic traits inventory – short version; GM, grandiose-manipulative; CU, callous-unemotional; II, impulsive-irresponsible. ^∗∗^*p* < 0.001. The correlations between the corresponding factors were in bold.*

As for the YPI-S, the Cronbach’s αs for the total score, GM, CU, and II were 0.786, 0.695, 0.545, and 0.758. The MIC values of the total and factor scores were all in the recommended range, from 0.174 (CU) to 0.340 (II). The inter-factor correlations of the YPI-S were *r*_*GM–CU*_ = 0.401, *r*_*GM–II*_ = 0.280, and *r*_*CU–II*_ = 0.356, and the correlations between the total score and factor scores were 0.700 (*r*_*Total–GM*_) to 0.777 (*r*_*Total–II*_). The correlations between the two versions of the YPI were *r*_*Totals*_ = 0.926*, r*_*GM*__*s*_ = 0.871, *r*_*CUs*_ = 0.864, and *r*_*II*__*s*_ = 0.887.

### Convergent and Criterion Validity

Table 8 displays the zero-order and partial correlations (i.e., controlling for the remaining factors of the YPI/YPI-S) with APSD-SR, BES, and RPQ. In terms of the convergent validity, the YPI/YPI-S total score, the GM, and the II showed significantly strong associations with the corresponding APSD total and factors at the zero-order (*r*s = 0.652/0.605, 0.547/0.454, and 0.688/0.661, *p*s ≤ 0.001) and partial correlation levels (*r*_*GM–Narcissism*_ = 0.377/0.346, *r*_*II–Impulsivity*_ = 0.579/0.608, *p*s ≤ 0.001). Conversely, the YPI/YPI-S CU correlated poorly with the APSD CU (*r*_*zero–order*_ = 0.137/0.075, *r*_*partial*_ = 0.003/−0.005). Relatively weaker correlations, especially the GM in relation to the APSD CU, were found in the un-corresponding factors. But the correlations of YPI/YPI-S II at the zero-order level with APSD CU were significantly acceptable (see Table 8). Moreover, the three YPI and YPI-S factor scores were significantly positively and moderately (CU) to strongly (GM and II) correlated to the APSD total score.

**TABLE 8:** Zero-order and partial correlations between YPI without reversed items/YPI-S scores and external variables.

	**GM**				**CU**			**II**			**Total score**		
		**YPI**	**YPI-S**	***z***	**YPI**	**YPI-S**	***z***	**YPI**	**YPI-S**	***z***	**YPI**	**YPI-S**	***z***
**APSD**													
Total	Zero	0.502^∗∗∗^	0.353^∗∗∗^	8.149^∗∗∗^	0.438^∗∗∗^	0.327^∗∗∗^	5.731^∗∗∗^	0.669^∗∗∗^	0.636^∗∗∗^	2.305^*^	0.652^∗∗∗^	0.605^∗∗∗^	3.929^∗∗∗^
	Part	0.224^∗∗∗^	0.204^∗∗∗^	0.992	–0.010	0.065	–3.540^∗∗∗^	0.524^∗∗∗^	0.574^∗∗∗^	–3.140^∗∗^			
Narcissism	Zero	0.547^∗∗∗^	0.454^∗∗∗^	5.294^∗∗∗^	0.377^∗∗∗^	0.318^∗∗∗^	2.984^∗∗^	0.465^∗∗∗^	0.426^∗∗∗^	2.271^*^	0.563^∗∗∗^	0.531^∗∗∗^	2.466^*^
	Part	0.377^∗∗∗^	0.346^∗∗∗^	1.617	–0.018	0.077	–4.487^∗∗∗^	0.240^∗∗∗^	0.320^∗∗∗^	–4.329^∗∗∗^			
Callous-Unemotionality	Zero	0.091	0.013	3.784^∗∗∗^	0.137^∗∗^	0.075	2.942^∗∗^	0.273^∗∗∗^	0.282^∗∗∗^	–0.485	0.202^∗∗∗^	0.183^∗∗∗^	1.238
	Part	–0.058	–0.066	0.388	0.003	–0.005	0.377	0.246^∗∗∗^	0.281^∗∗∗^	–1.880			
Impulsivity	Zero	0.385^∗∗∗^	0.262^∗∗∗^	6.355^∗∗∗^	0.427^∗∗∗^	0.321^∗∗∗^	5.447^∗∗∗^	0.688^∗∗∗^	0.661^∗∗∗^	1.940	0.604^∗∗∗^	0.580^∗∗∗^	1.924
	Part	0.021	0.070	−2.375^*^	0.044	0.093	−2.316^*^	0.579^∗∗∗^	0.608^∗∗∗^	–1.894			
**BES**													
Total	Zero	–0.080	–0.021	–2.861^∗∗^	–0.252^∗∗∗^	–0.201^∗∗∗^	−2.474^*^	–0.181^∗∗∗^	–0.187^∗∗∗^	0.316	–0.189^∗∗∗^	–0.191^∗∗∗^	0.130
	Part	0.106^*^	0.088	0.875	–0.211^∗∗∗^	–0.167^∗∗∗^	−2.115^*^	–0.074	–0.138^∗∗^	3.331^∗∗∗^			
Affective empathy	Zero	−0.102^*^	−0.108^*^	0.292	–0.273^∗∗∗^	–0.196^∗∗∗^	–3.747^∗∗∗^	−0.111^*^	–0.077	–1.766	–0.175^∗∗∗^	–0.166^∗∗∗^	–0.584
	Part	0.063	–0.032	4.605^∗∗∗^	–0.259^∗∗∗^	–0.161^∗∗∗^	–4.744^∗∗∗^	0.037	–0.003	2.069^*^			
Cognitive empathy	Zero	–0.014	0.091	–5.096^∗∗∗^	−0.104^*^	−0.108^*^	0.189	–0.173^∗∗∗^	–0.223^∗∗∗^	2.642^∗∗^	−0.113^*^	–0.127^∗∗^	0.901
	Part	0.103^*^	0.185^∗∗∗^	–4.019^∗∗∗^	–0.050	–0.093	2.033^*^	–0.162^∗∗∗^	–0.228^∗∗∗^	3.487^∗∗∗^			
**RPQ**													
Total	Zero	0.521^∗∗∗^	0.373^∗∗∗^	8.196^∗∗∗^	0.470^∗∗∗^	0.339^∗∗∗^	6.862^∗∗∗^	0.741^∗∗∗^	0.687^∗∗∗^	4.141^∗∗∗^	0.702^∗∗∗^	0.644^∗∗∗^	5.141^∗∗∗^
	Part	0.219^∗∗∗^	0.227^∗∗∗^	–0.398	–0.015	0.058	–3.444^∗∗∗^	0.617^∗∗∗^	0.632^∗∗∗^	–1.012			
Proactive aggression	Zero	0.528^∗∗∗^	0.397^∗∗∗^	7.306^∗∗∗^	0.459^∗∗∗^	0.336^∗∗∗^	6.411^∗∗∗^	0.697^∗∗∗^	0.624^∗∗∗^	5.194^∗∗∗^	0.683^∗∗∗^	0.619^∗∗∗^	5.523^∗∗∗^
	Part	0.249^∗∗∗^	0.263^∗∗∗^	–0.702	–0.010	0.062	–3.398^∗∗∗^	0.554^∗∗∗^	0.557^∗∗∗^	–0.189			
Reactive aggression	Zero	0.453^∗∗∗^	0.304^∗∗∗^	7.924^∗∗∗^	0.427^∗∗∗^	0.304^∗∗∗^	6.307^∗∗∗^	0.701^∗∗∗^	0.674^∗∗∗^	1.976^*^	0.641^∗∗∗^	0.595^∗∗∗^	3.800^∗∗∗^
	Part	0.137^∗∗^	0.140^∗∗^	–0.147	–0.017	0.041	–2.735^∗∗^	0.585^∗∗∗^	0.624^∗∗∗^	–2.579^∗∗^			

YPI, the Youth Psychopathic Traits Inventory; YPI-S, the Youth Psychopathic Traits Inventory – Short Version; GM, grandiose-manipulative; CU, callous-unemotional; II, impulsive-irresponsible; APSD, the self-report Antisocial Process Screening Device; BES, the Basic Empathy Scale; RPQ, the Reactive-Proactive Aggression Questionnaire; MIC, mean inter-item correlation. Results of the YPI total, CU factor and callousness subscale without the reversed items are presented after the tab. ^*^p < 0.05, ^∗∗^*p* < 0.01, ^∗∗∗^*p* < 0.001.

The criterion validity of the YPI/YPI-S was further supported by their relation with the BES and the RPQ (see Table 8). Overall, the YPI/YPI-S and BES revealed negative and mostly significant correlations at the zero-order level, yet the GM factors were marginally correlated with the BES. After controlling for the other two YPI/YPI-S factors (partial correlations), only the GMs showed modestly significantly positive relations with the BES cognitive empathy (*r*s = 0.103 and 0.185, respectively), as well as the YPI GM in relation to the BES total score (*r* = 0.106). Results also revealed that both the YPI and YPI-S positively and significantly correlated with the RPQ total score, as well as proactive and reactive aggression at the zero-order (*r*_*YPI*_ = 0.427–0.741; *r*_*YPI–S*_ = 0.304–0.687) and partial correlation level (*r*_*YPI*_ = −0.010 to 0.617; *r*_*YPI–S*_ = 0.041 to 0.632).

Notably, the correlation pattern of the original YPI and its short version were compared by calculating *Z* values (*p* ≤ 0.01, two-tailed for significance) which was based on Dunn and Clark’s (1969) method. Results showed that the strength of the correlations for some variables differed slightly between the YPI and the YPI-S.

## Discussion

This investigation aimed predominantly to validate the factor structure and psychometric properties of the YPI and YPI-S in a large sample of male Chinese detained youth. The results of CFA for the YPI at the item level revealed that the three reversed items of the CU factor had a problem with factor loadings as well as associations with other factors. In line with a previous study using a Chinese community sample (Wang et al., 2017), the bifactor structure of the YPI (removing the reversed items) at the subscale level and the bifactor structure of the YPI-S at the item level achieved an adequate fit to this data. Reliability analysis indicated the YPI and YPI-S total and factor scores had relatively satisfactory internal consistency. The YPI/YPI-S positively associated with APSD-SR and RPQ, but associated only modestly or non-significantly with the BES. Overall, the original YPI outperformed the YPI-S in psychometric measurements, regardless of limitations on assessment time.

### Factor Structure

Consistent with previous investigations into the factor structure of the YPI on the item level (Pihet et al., 2014; Wang et al., 2017), evidence obtained in our study indicated that the model fit for the one-factor model, the correlated three-factor model, the uncorrelated three-factor model, and the bifactor model were all inadequate. Examination into the factor structure of other self-reported psychopathic assessments had similar findings at the item level, as Wang et al. (2017) reviewed. As also found by Wang et al. (2017), excluding three reversed scored items from the CU factor did not improve the fitness for the correlated three-factor in detained juveniles. Further evidence also showed that the reversed scored CU items showed poor psychometric assessment, including low factor loadings and correlations with the other nine factors. This may be due to the influence of social desirability, positive phrase wording, or inaccurate translation of CU traits, rather than the specificity of the sample, as Wang et al. (2017) hypothesized, but this needs further examination. On the subscale level, abundant studies of the YPI across different samples have replicated a hierarchical three-factor structure (e.g., Declercq et al., 2009; Pechorro et al., 2015), yet this did not apply to the Chinese detained boys, and our findings confirmed the superiority of the bifactor structure in the original YPI scores. Such a bifactor model also performed better than other models when looking at the YPI-S scores in the current study’s sample, in concert with several empirical researches (Pihet et al., 2014; Zwaanswijk et al., 2016; Wang et al., 2017).

Remarkably for the YPI, 78.5% (ω_*H*_) of the variance of unit-weighted total scores can be attributed to the individual differences in the general factor (i.e., psychopathy), and only 11.6% (ω – ω_*H*_) of the reliable variance in total scores can be attributed to the multidimensionality caused by the three factors. Also, given that the ECV approached 0.70, the YPI general factor should be a unidimensional construct. Results of the YPI-S suggested that the ω_*H*_, as well as the ECV, gave more insight into a good indication of the general factor saturation. Namely a conceptualization of the multifaceted psychopathy construct was supported by the YPI-S, in line with prior findings (Wang et al., 2017).

### Internal Consistency and Inter-Correlations

Analysis of the internal consistency of the YPI suggests marginal to excellent Cronbach’s α of the YPI total scale and its three factors scores. Otherwise, there were general issues that the unemotional subscale and the callousness subscale revealed unacceptable α values (e.g., Declercq et al., 2009; Pechorro et al., 2015), which might at least in part reflect the small number of items of the subscales (van Baardewijk et al., 2010; Colins et al., 2012) or the insufficient representativeness of component items, and reverse coding items generally showed quite weak inter-item correlations (e.g., Pechorro et al., 2015). Deleting these items improved the reliability of the YPI total and the CU factor score, but caused extremely low reliability of the callousness score, likely because it was now measured by only two items. Regarding the MIC, the total score, the three factors, and all the subscales except callousness, these were all within the recommended benchmarks of 0.15 to 0.50, indicating adequate homogeneity between most items. As noted in previous studies (Colins et al., 2012; Pechorro et al., 2015), despite the removal of nearly two thirds of the items, analysis of the internal consistency suggested the YPI-S total scores and factor scores showed generally satisfactory reliability coefficients. Remarkably, a low α value for the CU score was also revealed. The affective factor of psychopathy referring to CU traits is difficult to assess, to some extent (Pihet et al., 2014). However, the YPI-S outperformed the original version of the questionnaire in measuring CU traits because the short version of the questionnaire offered a more internally consistent CU score, as reported in a study using Chinese community adolescents (Wang et al., 2017).

Similar to previous findings (van Baardewijk et al., 2010; Colins et al., 2012), mostly moderate to high statistically significant positive associations were yielded between the YPI total and its factors. The same pattern of associations was replicated in the YPI-S total and its factors, and the YPI-S showed a high convergence with the original YPI, which has been cross-validated in other investigations (e.g., van Baardewijk et al., 2010; Wang et al., 2017). Taken together, the YPI-S covered all core characteristics of the psychopathic personality construct, making it a psychometrically reliable tool for use in various settings or samples.

### Convergent and Criterion Validity

The convergent validity of the YPI/YPI-S with the APSD-SR and three conceptually corresponding factors revealed mostly moderate to high significantly positive correlations, which demonstrated the expected overlap. However, there was the exception of the CU factor of the YPI/YPI-S showing poor relations with the APSD-SR CU, also in accordance with findings reported in previous studies (e.g., Poythress et al., 2006; Colins et al., 2014). According to Wang et al. (2017), this finding was possibly interpreted as either a problem in the copy of the factor structure of the APSD-SR, or the poor performance of the YPI/YPI-S CU factor.

Regarding the criterion-related validity, the associations mostly revealed the expected null or negative correlations of psychopathy traits with empathy, consistent with prior research (Pechorro et al., 2017). The negative moderate-but-significant relations between the BES scores and the CU factor of the YPI/YPI-S indicated that the affective factor of psychopathy was characterized as a callous predisposition with a lack of empathy (American Psychiatric Association [APA], 2013). Generally, the partial (vs. zero-order) correlations were most often a bit weaker or less significant between the YPI/YPI-S factor scores and external variables, with a clear exception between YPI/YPI-S GM and cognitive empathy. Perhaps individuals with a high grandiose-manipulative trait like to identify, or even take advantage of others’ depression (cognitive measure) rather care about it (affective).

The associations with the RPQ continued to support the criterion validity of the YPI/YPI-S in the current study. Zero-order analyses revealed significantly positive and moderate to satisfactory correlations between the psychopathy factors of both the YPI and YPI-S and reactive and proactive aggression, confirming findings of previous studies (e.g., Colins et al., 2014, 2017). These significant correlations were found even after controlling for other psychopathy factors, except for the CU factor. Altogether, the abovementioned findings demonstrated the expected overlap with several facets of psychopathic personality and aggression (Poythress et al., 2006; Colins et al., 2014), and respondents self-reporting high psychopathic traits also tended to report higher levels of aggression (Blair, 2010; Colins et al., 2017).

Overall, the YPI-S has the same pattern of correlations as the original YPI, but in this particular study, these correlations were somewhat weaker. However, the YPI-S continues to show superiority in reliability analysis and assessment settings in comparison to the lengthy YPI. Furthermore, both versions of the YPI as clinical tools could help identify detained youths who show high levels of affective characteristics, allowing psychological staff to use different and more specific treatment approaches (Colins et al., 2017). For example, interventions aiming to improve emotion processing or regarding reward-oriented strategies (Frick, 2009) might be more effective in reducing aggression in detained boys showing psychopathic traits, given that antisocial juveniles with overt CU traits tend to have difficulty dealing with negative emotional stimuli and lack sensitivity to punishment cues (Fisher and Blair, 1998; Kimonis et al., 2008).

### Limitations

Some study limitations should be taken into account. First and foremost, the use of a male juvenile criminal sample precluded direct comparison between genders, and this is the only population that an inference could be drawn upon. In fact, there is evidence that female offenders express different aspects of the psychopathic personality disorder than we see in males (Pechorro et al., 2017), so future research should focus on female populations, test for potential gender differences in the validity and reliability of the YPI/YPI-S. Second, this investigation relied entirely on self-report information, which might result in shared method variance. Therefore, collateral information from family members, peers, and so on should be collected and considered in future research. On the other hand, psychopathic individuals tend to engage in response distortion styles (i.e., desirable responding and malingering) on self-report assessments (Ray et al., 2013). Further investigation should adopt additional stand-alone response style measures to detect deviant or socially desirable response styles. Third, a cross-sectional design may restrict conclusions on the predictive utility and causal inferences of YPI traits, thus we will attempt to conduct future longitudinal studies that evaluate correlations over time. Then in light of relatively poor psychometric properties (e.g., the low reliability, insufficient content validity) of callousness and unemotional subscales, the YPI is proposed to be revised in the future (Andershed et al., 2007) or to be used with complementary specific instruments measuring callous-unemotional traits. Finally, we suspect that the YPI’s validity is likely to be threatened by its positively worded items, as adolescents are inclined to admit to these items even if they are non-psychopathic. This possible influence of the item phrasing should be addressed in the future.

## Conclusion

Overall, the current study replicated a bifactor model among Chinese detained boys at the subscale level for the YPI, and at the item level for the YPI-S. The YPI and YPI-S presented marginal to good internal consistency. The correlations with a wide range of external variables expanded the convergent and criterion validity of the YPI and YPI-S, but the relations to external validity correlates of the YPI-S are generally somewhat weaker than those of the YPI. Altogether, this investigation generally lends support for the usefulness of both the YPI and YPI-S to assess psychopathic traits in juvenile Chinese detained males.

## Data Availability

The datasets generated for this study are available on request to the corresponding author.

## Author Contributions

WY, XZ, CZ, and JL made substantial contribution to the analysis and interpretation of the data, drafted the manuscript, provided final approval of the version to be published, and agreed to be accountable for all aspects of the work in ensuring that questions related to the accuracy or integrity of any part of the work are appropriately investigated and resolved. M-CW and YG made substantial contributions to the conception and the design of the study, drafted the manuscript, provided final approval of the version to be published, and agreed to be accountable for all aspects of the work in ensuring that questions related to the accuracy or integrity of any part of the work are appropriately investigated and resolved.

## Conflict of Interest Statement

The authors declare that the research was conducted in the absence of any commercial or financial relationships that could be construed as a potential conflict of interest.

## Footnotes

1 Using a spreadsheet which was developed from DeCoster and Iselin (2005) and can be searched at: http://stat-help.com/spreadsheets.html.

2 Mplus warned that the residual covariance matrix at the YPI subscale level is not positive definite. Notably, the Remorselessness subscale showed a deviant factor loading (see Table 5), which could be attributed to a negative residual variance. To address this concern, MLR was replaced with a robust Maximum Likelihood estimator (MLM), while also using the method of unfixed variance, but both failed. Even though the current study reported the total results, the ω and ω_*H*_/ω_*HS*_ of the original YPI bifactor model were not computed. In follow-up investigations, we tried to remove several items to explore more acceptable models.
